# Thermodynamics of CuPt nanoalloys

**DOI:** 10.1038/s41598-018-27308-1

**Published:** 2018-06-14

**Authors:** K. Rossi, L. B. Pártay, G. Csányi, F. Baletto

**Affiliations:** 1 Physics Department, King’s College London, London, WC2R 2LS United Kingdom; 2 Department of Chemistry, University of Cambridge, Cambridge, CB2 1EW United Kingdom; 3 Department of Chemistry, University of Reading, Whiteknights, Reading, RG6 6AD United Kingdom; 4 Engineering Department, University of Cambridge, Cambridge, CB2 1PZ United Kingdom

## Abstract

The control of structural and chemical transitions in bimetallic nanoalloys at finite temperatures is one of the challenges for their use in advanced applications. Comparing Nested Sampling and Molecular Dynamics simulations, we investigate the phase changes of CuPt nanoalloys with the aim to elucidate the role of kinetic effects during their solidification and melting processes. We find that the quasi-thermodynamic limit for the nucleation of (CuPt)_309_ is 965 ± 10 K, but its prediction is increasingly underestimated when the system is cooled faster than 10^9^ K/s. The solidified nanoparticles, classified following a novel tool based on Steinhardt parameters and the relative orientation of characteristic atomic environments, are then heated back to their liquid phase. We demonstrate the kinetic origin of the hysteresis in the caloric curve as (i) it closes for rates slower than 10^8^ K/s, with a phase change temperature of 970 K ± 25 K, in very good agreement with its quasi-thermodynamic limit; (ii) the process happens simultaneously in the inner and outer layers; (iii) an onion-shell chemical order - Cu-rich surface, Pt-rich sub-surface, and mixed core - is always preserved.

## Introduction

The intimate interdependence of structural - size, geometrical shape, chemical composition, and elemental ordering - and chemophysical - catalytic, plasmonic, and magnetic - properties generates a fundamental interest in elucidating the thermodynamic behaviour of bimetallic nanoalloys (BMNAs)^1–6^. Finite size effects in BMNAs cause the breakdown of classical thermodynamics due to the significant surface contribution to the energy and entropy of the system^7–11^. Resolving the complexity of phase changes in nanoclusters is a compelling and open problem, both from a theoretical and an experimental perspectives^12–14^. Elucidating the thermal behaviour of nanosized systems will help to understand whether structural heterogeneity can be explained and hence exploited^15–17^. An in depth understanding of the freezing transition is of great appeal as this is key to one widespread nanoparticle formation process: the one taking place in sources where a mixture of metallic vapour and inert gas is first injected into a cooling chamber, then condensed into liquid droplets, and finally subject to a supersonic expansion in vacuum. The so-formed structures, although selected in size, might be morphologically poly-disperse and could display exotic structural features such as multiple five-fold axes, single or multiple fcc tetrahedral subunits and surface defects^14,18–23^.

Many studies have been devoted to characterise distinctive phenomena of melting and nucleation (solidification) at the nanoscale, such as size-dependent melting point depression^24^, phase coexistence^25^, surface-melting^26^, chemical ordering evolution^27–29^, superheated and supercooled phases^30^, and melting-freezing hysteresis^22,31–33^. Phase changes can be numerically investigated with atomistic resolution to understand the thermal contribution during morphological transitions at the nanoscale. A numerical tool commonly used for these tasks is Molecular Dynamics, with the main caveat that the accessible temperature change rates, which span from 10^8^ K/s to 10^13^ K/s^19,33,34^, are faster then the typical experimental values of ∼10^4^ K/s^21^. One of the main difficulties in modeling the solidification and the melting of mono- and bi-metallic nanoparticles lies, therefore, in untangling whether the observed hysteresis between the two processes - melting and freezing temperatures can indeed differ at the nanoscale^22,31–33^- is an intrinsic property of the considered nanosystem or an effect merely driven by kinetic factors, due to the reduced time-scale of in-silico experiments, which could be not long enough to have a representative sampling of the cluster’s free energy landscape. The difference in the pathways from solid to liquid and from liquid to solid, or the appearance of superheated and supercooled phases dictated by the finite time scale of numerical simulations and experiments, can both be at the origin of the opening of an hysteresis in the caloric curve. So far, the lack of studies directly comparing nucleation and melting in BMNAs, the intrinsic difficulty of exactly defining - if possible at all - a thermodynamic limit, and - in the affirmative - how to characterise it, has hindered our understanding of the nano-thermodynamics of metallic nanoalloys.

## Computational Methods

In the present work, we compare results from an iterative temperature molecular dynamics (itMD)^35–37^ and Nested Sampling (NS)^38–44^ numerical simulations to characterise the freezing and the melting process of CuPt BMNAs with a 1:1 stochiometry, which have recently gauged interest as promising catalysts^45–49^. In the rest of the manuscript, we use freezing, cooling, nucleation or solidification as synonyms. Similarly, the terms liquid and melted are used to describe the same phase. We fix the size of the cluster under investigation at 309 atoms, a nuclearity which is not necessarily a magic number for considerably mismatched (8%) BMNAs, but is sufficiently large to avoid peculiar effects appearing in the sub-nano regime; to clearly distinguish inner and outer shells; to address trends in the chemical ordering in both the liquid and solid phases; and to allow for the comparison of itMD and NS statistics at a reasonable computational cost. Metal-metal interactions are modelled following the second moment approximation of the tight binding theory, in the Rosato-Guillope’-Legrande formulation^50^, and their parametrisation is taken from ref.^32^. The choice of a tight binding potential in second-moment approximation (TB-SMA) to describe interatomic interactions is dictated by the long time and length scales involved in our calculations. A preliminary and positive validation of this potential for the case of a 55 atoms CuPt nanoalloy is discussed in the SI. We here just recall that on one hand TB-SMA potentials are considered reliable for calculating kinetic and thermodynamic properties of cluster larger than tens of atoms, on the other we are aware that complex quantum effects, e.g. charge transfers, are not accounted by this approximation.

itMD consists of concatenated loops where the temperature is increased by the quantity Δ*T* every Δ*τ* time so that the kinetics of the phase change can be analysed in terms of a unique parameter, named as temperature change rate, *λ* = Δ*T*/Δ*τ*. itMD encodes information on representative trajectories at finite temperatures, allows to estimate caloric curves, and to detect shape fluctuations occurring in a time scale comparable with Δ*τ*^19,35^. In our simulations, Newton’s equations of motion are integrated using a velocity Verlet with a time step of 5 fs and the temperature is controlled by an Andersen thermostat with a frequency of 10^11^ Hz to mimic the presence of an inert gas, acting as a thermal bath, surrounding the cluster. Previous studies have shown that the selected frequency do not alter adatom diffusion properties^51^. Keeping fixed Δ*T* = 25 K, we vary Δ*τ* = 1, 2.5, 5, 25, 250 ns with *λ* spanning over two orders of magnitude. A comparison with simulations employing similar *λ* values but Δ*T* = 25, 10, 5 K and Δ*τ* = 1 ns can be found in the SI. We perform at least 25 independent runs for every *λ*. In nucleation calculations, the initial droplet is obtained after 10 ns of a microcanonical run at 1400 K, while every solidified structure is then heated up back to their liquid phase.

Nested sampling is a global sampling algorithm to estimate the density of states^38–44^. It provides an unbiased exploration of the energy landscape of the system while taking into account the entropic contribution and determining the relative phase space volume of different basins without *a priori* knowledge of solid structures. A detailed description of the algorithm is presented in the SI, let us here just mention that the sampling of the density of states allows the evaluation of the partition function at arbitrary temperatures. Further, as the sampling procedure itself is independent of temperature, the partition function can be evaluated *a posteriori* at any temperature and from the former other thermodynamic variables, such as the internal energy, can be evaluated as a function of temperature. Nested sampling calculations are performed as described in ref.^40^. We use 1920 configurations in our sample set, and initially these are generated such that the 309 atoms are randomly placed in a periodic cubic box of volume of 91125 Å^3^. In order to demonstrate the convergence of our results, seven parallel runs are performed, five with 4.8 × 10^4^ steps at every nested sampling iteration, and further two with 9.6 × 10^4^ steps. 92% of these are single atomic displacements, the rest being swaps of different atomic species. Notwithstanding the large size of the cluster and limits in computational resources which lead to a compromise in the sample size used in the calculations, two of the NS runs explored all the structural motifs of interest. Hence, we discuss the results of these two runs in the paper, while statistics from the remaining five are reported in the SI.

### Structural Characterization

The comparison between these two numerical tools enables the investigation of how the kinetic contributions influence the formation process, including the analysis of the probability to end into a given morphological family. On this regard, we introduce a novel structural characterisation scheme based on the relative arrangement of five-fold axes and fcc-grains in the cluster. Unlike bulk materials, nanometer-sized clusters exhibit various distinct structural motifs, which are generally classified according to the relative occurrence of characteristic local environments, yet not taking into account their relative arrangement. Especially at temperatures close to melting, nanoparticles exhibit less symmetrical shapes and strongly defected surfaces, with broadened pair distance and angular distribution functions. These factors make it more difficult, if not misleading, to identify the geometry solely by calculating the ratio of atoms with a given characteristic order parameter of their local environment.

To determine which class a configuration belongs to, we first calculate the atomic *Q*_6_ and *W*_6_ Steinhardt^52^ bond order parameters and identify the atoms being an icosahedral centre (*Q*_6_ = 0.6633, *W*_6_ = −0.1697), part of a five-fold axis (*Q*_6_ = 0.4312, *W*_6_ = −0.09351), or belonging to sheets of hcp (*Q*_6_ = 0.48476, *W*_6_ = −0.012442) or fcc (*Q*_6_ = 0.57452, *W*_6_ = −0.01316) layers. This choice is not only helpful to our characterisation scheme, but these environments are often the first to be easily distinguishable in solidified clusters and possibly nucleation starts from these too^22^. Next we calculate the angles between the five-fold axes, in the eventuality that more than one is present, and check the arrangement of hcp layers if applicable. We then classify each nanoparticle into three morphological families:

1. Closed packed (Cp): clusters solely formed by stacked close-packed layers, where there are no five-fold symmetry axes. All morphologies obtained by cutting an fcc lattice, as arising from the truncation of an octahedron, such as cuboctahedra, or an hcp crystal, belong to this class.
2. Decahedra (Dh): clusters where a single five-fold symmetry axis is present, though this structural feature does not have to necessarily contain the center of mass of the cluster. Marks- and Ino-decahedra are example of closed-shell structures which belong to this class.
3. Icosahedra (Ih): clusters where more than one five-fold symmetry axes is present, and intersect in one or more points, though this intersection can be off-centre or even found outside the cluster itself. Nanoparticles built up by multiple tetrahedral subunits, as closed-shell icosahedra as well as poly-icosahedra^51^, belong to this class.

**Figure 1:**
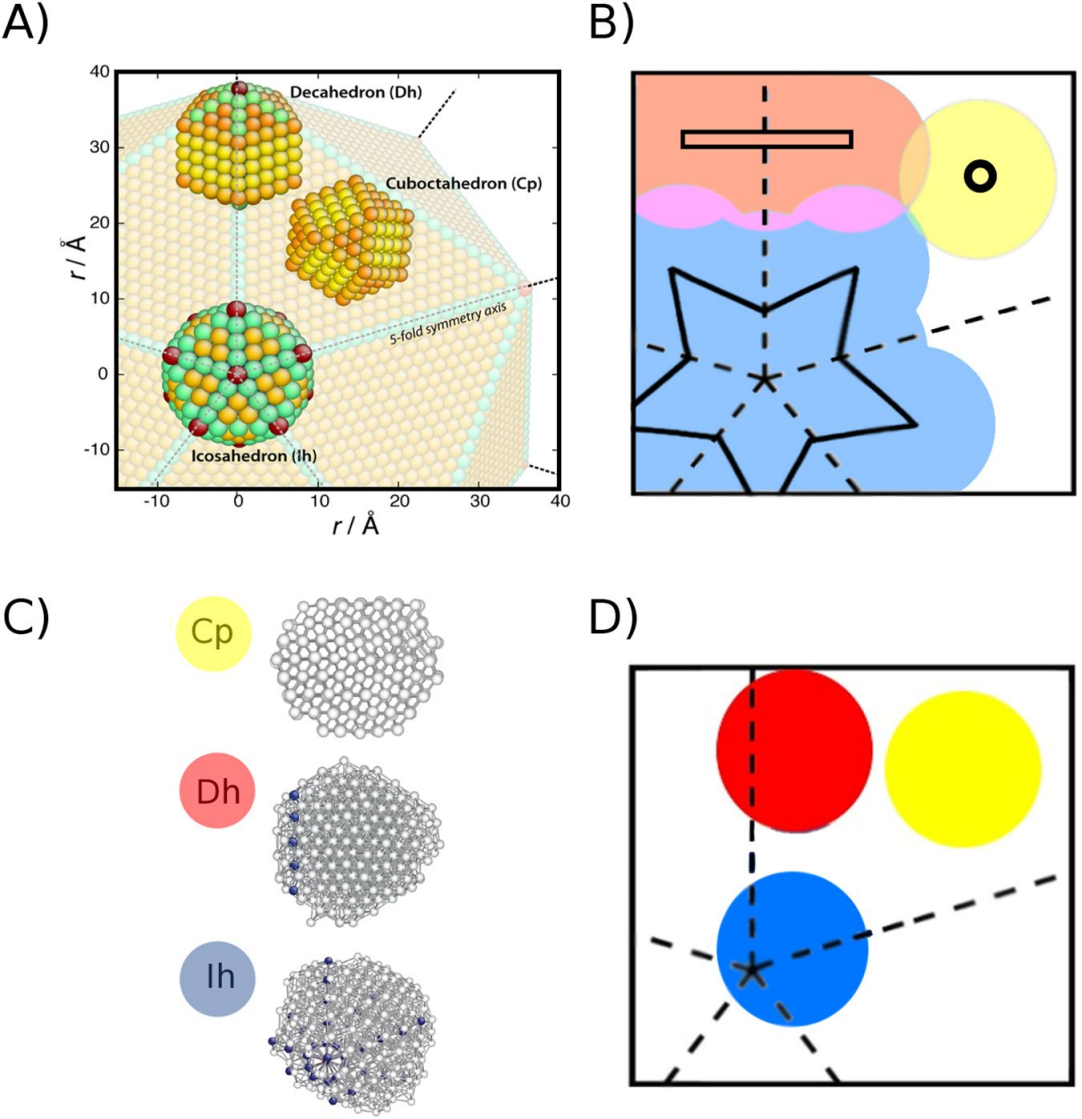
(**A**) Visual mapping of a 309 atoms closed-shell polyhedra into a 37995-atoms giant Ih. Though the angles between boundaries or arrangements of facets can be somewhat different from the exact values of that in a perfect polyhedron: a smaller icosahedron can be readily placed in the innermost shells of a larger one; a decahedron can be formed from a section of an icosahedron along one five-fold symmetry axis; a cuboctahedron from the close-packed tetrahedral space between the gIh five-fold boundaries. (**B**) 2D-projection of the g-Ih where the coloured regions label the three morphological families where each cluster, represented by a circle, falls in. Circles might overlap, as in the pink, orange, and green regions, but this classification depends only on the position of the circle-centre. The centre of Dh lie in the red rectangle, the Cp’s one in the yellow circle, and the Ih’s centre within the blue star, respectively. (**C,D**) Example of CuPt-nanoparticles obtained during the solidification process in itMD where atoms belong to a five-fold axis are in blue, icosahedral centre in red, otherwise in white, independently of their chemical species; their corresponding representation onto the 2D-gIh map.

To readily visualize the morphologies observed during NS and itMD calculations, we map the solidified clusters onto a much larger closed-shell icosahedron, referred to as giant icosahedron (gIh). According to the above classification, each cluster will fall in a specific region of the gIh, as identified by a different colour in Fig. 1A. The size of the gIh is lower bounded by the smallest one where perfect geometrical shapes belonging to the three listed morphological classes can be univocally inscribed, for the Cu_162_Pt_147_ case, a 37955 atoms (20 nm) gIh is needed. We then do a 2D-projection, where dashed lines represent the five-fold symmetry axes of the gIh upper half, and the solidified nanoparticles - examples taken from itMD simulations in Fig. 1B - are drawn as a circle with diameter reproducing the maximum pair distance in the cluster. The positions of those circles on the the 2D gIh (see Fig. 1C) follows from the relative arrangement of the center of mass of the nanoparticle, and the icosahedral center or the five-fold axis, if any. A circle corresponding to an Ih is positioned by using the distance between its center-of-mass and the closest icosahedral atom - either it is truly present in the cluster or calculated from the intersection of the five-fold axes - and aligning the longest five-fold axis of the cluster to one of the gIh. For Dh, the circle is located so that the distance between its center and the gIh five-fold axis approximates the one between the nanoparticle center-of-mass and its own five-fold axis.

## Results and Discussions

**Figure 2:**
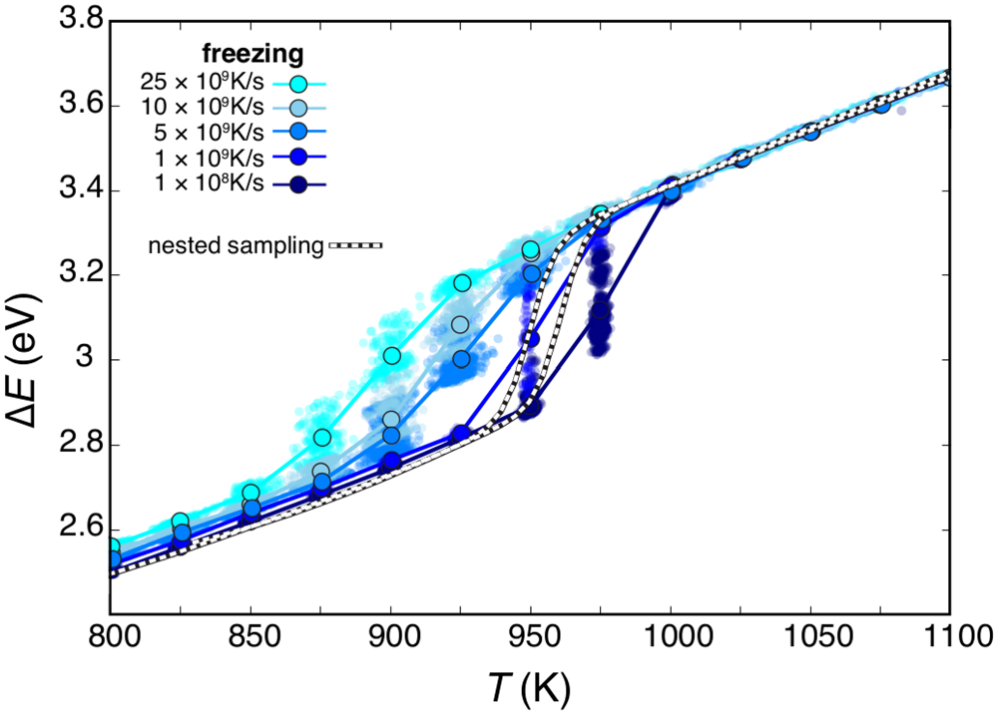
Caloric curves for the nucleation of a Cu_162_Pt_147_ liquid droplet: scattered points and filled circles refer to the itMD data averaged as discussed in the text, lines are guides to the eye only. Colours refer to *λ*, where a lighter blue tone corresponds to faster rate. NS results are shown by black and white dashed lines, and they should be considered as quasi-thermodynamic limit for that system.

To unravel the kinetic effects in phase change processes of Cu_162_Pt_147_, we analyse the caloric curves during nucleation as obtained from itMD and NS simulations, shown in Fig. 2. Here we plot the excess energy Δ*E*(*T*), calculated as the energy difference between the bulk and an equivalent number of atoms in their homo-metallic bulk counterparts, normalised with respect to the volume-surface contribution:

1 $${\rm{\Delta }}E(T)=({E}_{{\rm{tot}}}-({N}_{{\rm{Cu}}}{E}_{{\rm{Cu}}}^{{\rm{coh}}}+{N}_{{\rm{Pt}}}{E}_{{\rm{Pt}}}^{{\rm{coh}}}))/({N}_{{\rm{Cu}}}+{N}_{{\rm{Pt}}}{)}^{\mathrm{2/3}},$$

where *E*_tot_ is the total energy of the system, *N*_Cu_ and *N*_Pt_ are the number of Cu and Pt atoms in the cluster, and ${E}_{{\rm{Cu}}}^{{\rm{coh}}}=3.65$ eV and ${E}_{{\rm{Pt}}}^{{\rm{coh}}}=5.84$ eV are the bulk cohesive energies. While solid and liquid states are detected by the evolution of structural order parameters such as pair correlation, root-mean square displacement, common neighbour analysis, and bond-order parameters -as reported in SI- the estimate of the phase change temperature, T_*pc*_ is accomplished by fitting splines on the caloric curve, Δ*E*(*T*), and identifying the inflection points. In the NS framework, T_*pc*_ is estimated to be 965 ± 10 K, and it can be considered as the quasi-thermodynamic limit. Per each *λ* in itMD simulation, we calculate the cluster instantaneous temperature and excess energy and we report their average over the ensemble of our independent simulations (scattered points in Fig. 2). Successively we sum all the points corresponding to the same temperature interval (Δ*τ* long) and we average over that time (filled circles in Fig. 2). The error on ensemble and time averaged *T* and Δ*E* are almost negligible, except for Δ*E* in the phase change region (as discussed in SI). In the case of freezing itMD, depending on the cooling rate, the nucleation temperature shifts from T _*pc*_ = 888 K (*λ* = 25 K/1 ns) to T _*pc*_ = 975 K (*λ* = 25 K/250 ns), the latter falling within the NS prediction.

All solidified structures found by NS and a sample of a different temperature per itMD run, for a total of a minimum of one hundred per rate, are characterised according to our novel taxonomy tool. Table 1 summarises the structural population distributions right below the freezing point following a itMD or NS procedure. Both methods agree that more than the 80% of the nucleated structures show a twinned motif, with a minority of Dh, and only the 20% corresponds to Cp. The larger proportion of Ih over Dh clusters in itMD with respect to NS calculations suggests the presence of kinetic effects related to the formation of surface defects, including islands on hcp sites, or of shorter five-fold axis. Nonetheless, we note that the occurrence of the morphological groups is not excessively influenced by *λ* during itMD cooling simulations. Our general observations on the morphology of solidified clusters as both arising from NS and itMD, are the following.

**Table 1:** Percentages of the three morphological families as found in itMD freezing simulations at different *λ* and NS, taken just below T_*pc*_.

*λ* (10^9^ K/s)	Ih	Dh	Cp
0.1	75	7	18
1	73	10	17
5	69	12	19
10	75	14	11
25	72	8	20
NS (850–900 K)	58	18	24
NS (850–900 K)	54	22	24

The itMD occurrence corresponds to temperatures at 700 K, below which no morphological changes are observed, and they are averaged over all the 25 independent runs. The nested sampling percentages are reported for the two runs exploring all basins.

When more than one five-fold axis is present within the cluster, they are always arranged so that the angles between them are approximately 60–65°, close to the reference value in a perfect icosahedron (63.43°). The intersection of those axes can, eventually, lie outside the cluster (see SI). A large number of configurations with three or more five-fold axes, form triangles and tetrahedra, in agreement with ref.^22,34^, but we never observe structures with parallel five-fold axes -as shown for Au _923_-^21,53^, probably because the size of our clusters is significantly smaller. When three or more five-fold axes intersect in different points, they form triangles with edges 8–9 atoms long. On the other hand, if during the nucleation the cluster builds tetrahedral pieces, they have 5 (as expected in a perfect closed-shell Ih of 309 atoms) or 6 atoms in their edges. Dh often display their five-fold axis off-centre, which sometimes lies at the very surface of the cluster, with one of the nearest neighbours of the atoms missing (see SI). When there are no five-fold axis, the cluster is likely to show 8 or 9 close-packed layers, similarly to a perfect cuboctahedron which presents 9 fcc layers. In agreement with energetic considerations on hcp-island formation on monometallic nanoparticles^54^, the cluster sampled, however, always presents hcp layers too. During the formation of Cp shapes, we foresee kinetic contribution as the ratio of hcp to total layers decreases from 50% to 33% when *λ* is reduced, in agreement with NS which always predicts a fcc layers occurrence in the 50–70% range.

**Figure 3:**
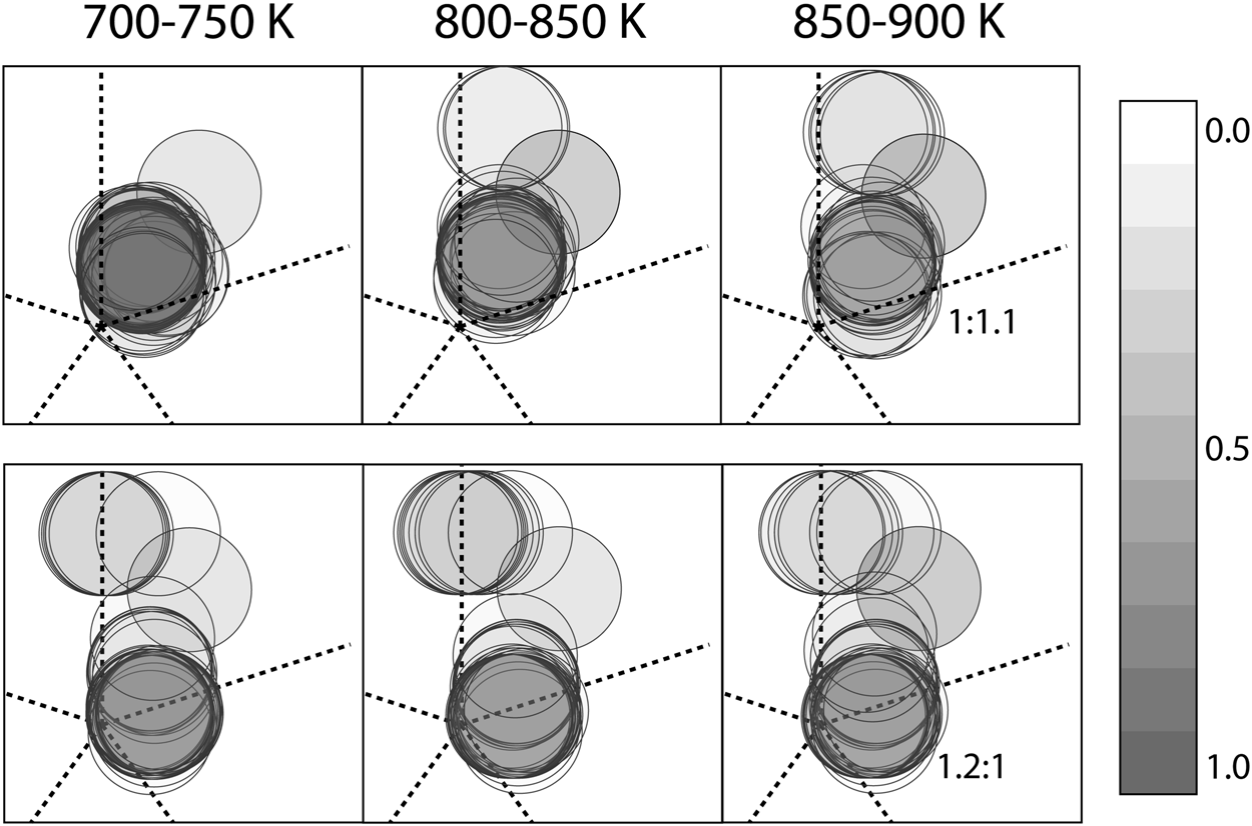
Projection of the sampled structural motifs onto the 2D-map of a gIh, as seen in the two parallel NS calculations at different temperatures. Darker shades indicate higher probability of the given geometry.

Looking at temperatures below the melting, we remark the ability of NS to explore simultaneously different morphological basins notwithstanding the large phase space of the system under investigation. We also highlight that during NS runs the ratio of the different morphologies changes with decreasing temperature (see Fig. 3), with the least occupied basins becoming even less populated and the structural basin being the most populated at high temperature staying to be the dominant one throughout the sampling (see SI). Contrasting this with itMD results, we can estimate the time scale of shape rearrangements in a CuPt_309_ clusters. Indeed we have that for cooling rates faster than 10^8^ K/ns only one basin of the landscape is visited, and no morphological structural transitions are observed below the solidification temperature. At *λ* = 10^8^ K/s, the itMD trajectory visits multiple basins - either Cp, Dh, or Ih as discussed in the SI - down to 780 K. Afterwards, the system remains trapped in one basin and with no morphological transitions occur, but still surface diffusion could take place. This result suggests that the characteristic observation time scale for shape rearrangements are larger than 25 ns for temperatures in the 750–950 K and more then ten times longer below this range.

**Figure 4:**
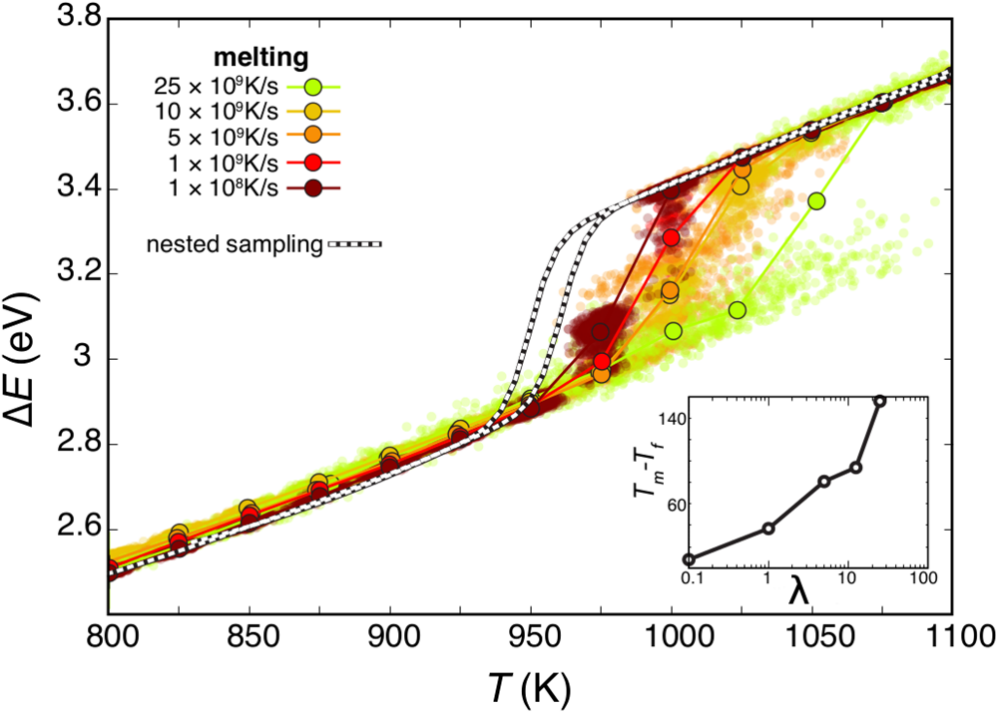
Melting caloric curve the previously solidified Cu_162_Pt_147_ by itMD, scattered points and filled circles as in Fig. 2. Colours refer to *λ*, where a lighter red tone corresponds to faster rate. NS results (black and white dashed lines) are reported as reference point. The inset shows the temperature-width, between the highest temperature at which the cluster is always solid during heating, T_*m*_, and T_*f*_, the lowest temperature at which nanoalloys are liquid during cooling, against *λ*.

To understand the nature of the hysteresis in the caloric curve we reverse the itMD procedure to melt the nanosystem again. When the ensemble of BMNAs found at the end of each itMD freezing run is heated up back to be a liquid droplet, we observe that the hysteresis in the caloric curve depends only on the chosen *λ*, as reported in Fig. 4. As the latter is reduced, the melting-freezing caloric curve hysteresis becomes considerably narrower: while *λ* = 25×10^9^ K/s shows a 160 K difference between the freezing and melting temperatures, it is of 80 K with *λ* = 5×10^9^ K/s and less than 15 K at *λ* = 10^8^ K/s, suggesting a closure of the gap at even slower rates. We stress that for this *λ* value, above 1000 K, Cu_162_Pt_147_ is always liquid while below 950 K is only solid. We expect at such small sizes a smooth-transition, and we claim that the temperature range where the phase change happens is between 950 K and 1000 K, independently of the initial shape. If we take, as before, the inflection points, the itMD estimate for the phase change of Cu_162_Pt_147_ is 970 K, in very good agreement with NS. The melting temperature of the cluster is depressed both respect to the bulk value for each single compound, ${{\rm{T}}}_{{\rm{Cu}}}^{m}$ = 1356.2 K, ${{\rm{T}}}_{{\rm{Pt}}}^{m}$ = 2047.2 K^55^ and of an alloy of similar chemical composition, ${{\rm{T}}}_{{\rm{CuPt}}}^{m}\sim$ 1844 K^56^, which is higher than a mere average. According to preliminary results on monometallic nanoclusters using itMD, the phase change temperature of a pure Cu _309_ cluster is 870 ± 20 K, while for a pure platinum cluster at the same size and shape, the transition is estimated to occur at 1225 ± 25 K.

**Figure 5:**
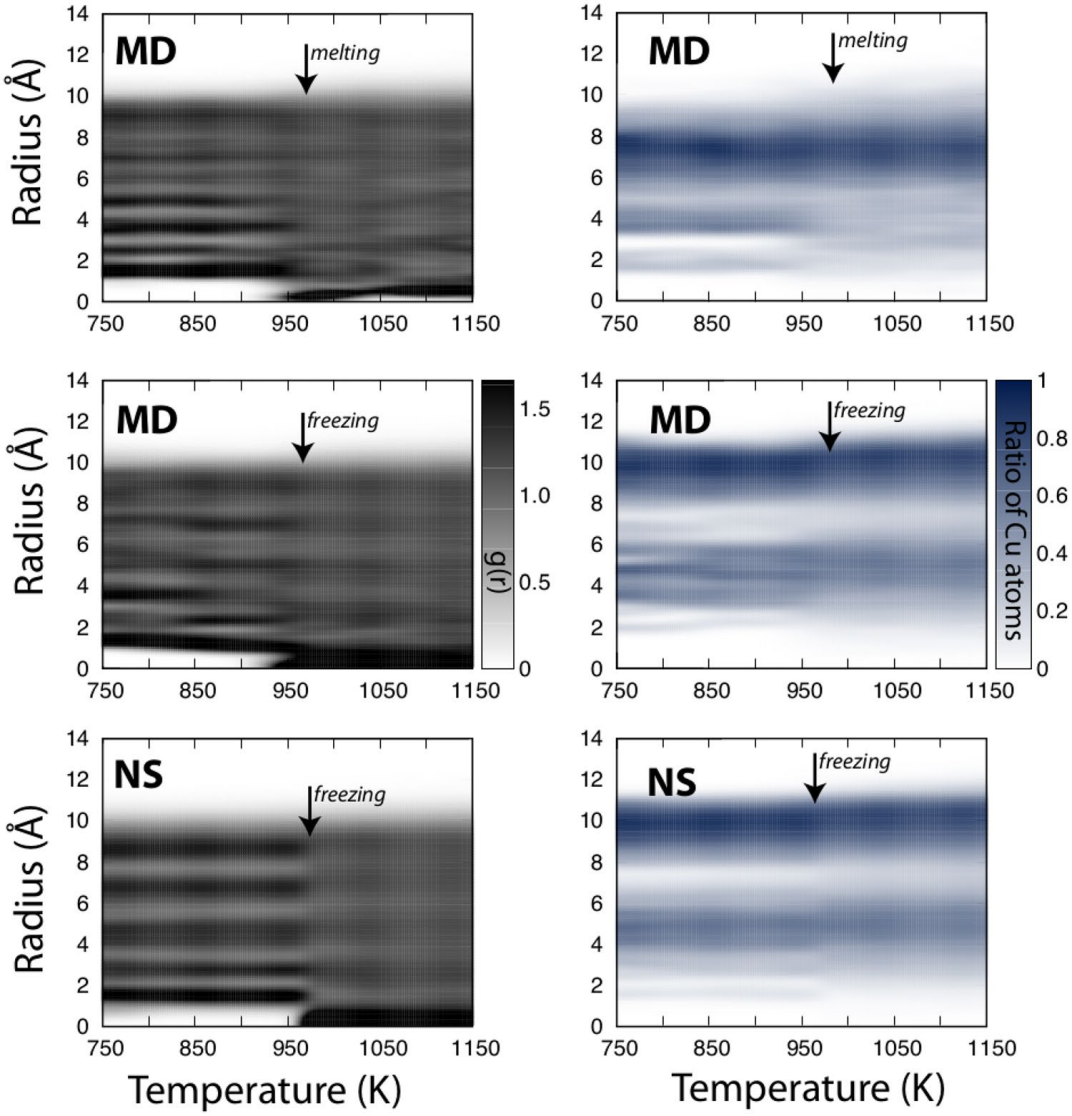
Radial distribution of atoms around the centre-of-mass as a function of temperature. The top two panel shows an example itMD run with a *λ* = 10^9^ K/s for melting and freezing, respectively, while the bottom panel shows the results from NS calculations. The left hand side panels show the overall distribution of atoms, while the right hand side figures show the relative occurrence of Cu atoms within the cluster. The estimated phase change temperatures are marked by arrows for reference.

Analysing the temperature dependence of the atomic radial distribution from the centre-of-mass of each nanoparticle, as reported in Fig. 5 left panel, for representative NS, itMD freezing, itMD melting, respectively, we can elucidate the mechanism throughout phase changes take place. At temperatures close to the critical one, both the surface and the inner layers of the cluster simultaneously show regions with liquid-like and a solid-like arrangement, with the interface between the two regions being either curved or flat (see SI). This observation suggests the absence of surface pre-melting or inner core solidification. Previous works on smaller (55 atoms) or considerably larger (1000–10000 atoms) mono- and bi-metallic systems^31,33,57,58^ shown that if the kinetics of the phase transition is different, for example, melting starts at the surface, while freezing takes place through the nucleation of a solid seed in the inner layers of the cluster, the hysteresis cannot be overcome by tuning the heating rate, as also observed in experiments^59,60^. Our results demonstrate that Cu_162_Pt_147_ have the same mechanisms for solid to liquid and liquid to solid transitions, and do not necessarily display a hysteresis between the melting and the freezing curves. This finding is in very good agreement with the size-behaviour expected in Ag nanoparticles which predicts the formation of a quasi-liquid layer, or, more in general, a surface pre-melting only at sizes larger than several nanometers^61,62^. Similarly, we can think that the interface energy required to create a quasi-liquid layer in 1–2.5 nm CuPt nanoparticles could be too big. Hence, the melting and the solidification follow the same mechanism and the associated phase change temperature coincides, in the condition that they proceed slowly enough. We would like to remark that comparing NS data, which provide an estimate at the quasi-thermodynamic limit, and MD simulations for the first time we have estimated this time scale to be 1 K/ns. Preliminary results show that this observation is preserved regardless the relative Cu concentration in the nano-alloy.

When probing the temperature evolution of the chemical radial distribution function, as in Fig. 5 right panel, we identify an onion-shell elemental distribution, both according to itMD and NS calculations: the outermost shell contains two thirds of the total copper population; the subsurface is Pt-rich (hitting the $\sim \mathrm{80 \% }$); the third and fourth layers have a 60% of Cu and 40% Pt, respectively. This onion-shell chemical ordering is preserved above the melting point and high temperature NS configurations show that it is the most favourable up to 2500 K, supporting the idea that it should be considered as the equilibrium chemical arrangement, in agreement with previous in-silico^32,63^ and *in-vitro*^48^ studies. It is interesting to note that in correspondence of the melting transition the radial distribution function identifies an atom at the center of the cluster, while earlier the center of mass of the system felt in between atoms. Nonetheless, others chemical patterns, for example following a Cu-surface dealloying, could exhibit a sufficient long life-time in agreement with previous theoretical^29,35^ and experimental studies^45,47,49^. Indeed, choosing a Pt-core Cu-shell nanoparticle as initial configuration in microcanonical MD simulations at room temperature, no reordering takes place within 100 ns. On this time scale, partial and strong elemental diffusion towards the most energetically favourable multi-shell arrangement starts at 600 K and 900 K, respectively (see SI).

## Conclusion

In conclusion, we studied the solid-liquid and liquid-solid phase change in a CuPt nanoalloy by two synergic computational tools, iterative Molecular Dynamics and Nested Sampling. We observed that the hysteresis in the caloric curve gradually narrows slowing the heating/cooling rate, *λ*, and closes when *λ* is less than 10^8^ K/s, providing an estimate of the phase change temperature, T_*pc*_, of 970 K ± 25 K. This result serves as an indirect proof that the itMD procedure employed to model nanoparticles freezing and melting can reproduce the quasi-thermodynamical limit, estimated to be 965 ± 10 K by NS calculations for Cu_162_Pt_147_. The closure of the hysteresis is rationalised in terms of the similar phase change mechanism occurring during the solidification and the liquefaction transitions, with no evidence of surface pre-melting or inner core initial nucleation.

The variety of shapes sampled after nucleation is classified into three families close-packed, decahedral and icosahedral, following a novel scheme, based on Steinhardt’s bond order parameters and the orientation of local atomic arrangements. This new scheme allows an intuitive visualisation of closed-shell, crystalline, low-symmetric, and exotic structures in reference onto the 2D-projection of a giant icosahedron. Our work might shed lights on the generation of twinned morphologies, the appearance of structural heterogeneity and how a thermodynamic control can be exploited for the rational design of nanoalloys. Indeed, we find that, during nucleation, Ih are the most abundant shapes with only a weak dependence of the morphological distribution on kinetic factors, tunable by *λ*. Nonetheless, *λ* affects considerably the structural evolution of nanoalloys below the nucleation temperature, with shape fluctuations observable within few tens of nanoseconds in the 750–950 K temperature range, and above the hundreds of nanoseconds for lower temperatures. The competition among various structural basins just below T_*pc*_, suggests high activation energy barrier for solid-to-solid transitions leading to very heterogeneous samples with diverse structural, and hence physicochemical, properties, that must be reckoned to tailor nanoalloys for target advanced applications.

## Electronic supplementary material

Thermodynamics of CuPt nanoalloys
