# Author Correction: Thermodynamics of CuPt nanoalloys

**DOI:** 10.1038/s41598-018-29408-4

**Published:** 2018-08-07

**Authors:** K. Rossi, L. B. Pártay, G. Csányi, F. Baletto

**Affiliations:** 1 Physics Department, King’s College London, London, WC2R 2LS United Kingdom; 2 Department of Chemistry, University of Cambridge, Cambridge, CB2 1EW United Kingdom; 3 Department of Chemistry, University of Reading, Whiteknights, Reading, RG6 6AD United Kingdom; 4 Engineering Department, University of Cambridge, Cambridge, CB2 1PZ United Kingdom

Correction to: *Scientific Reports* 10.1038/s41598-018-27308-1, published online 14 June 2018

The original version of this Article contained a typographical error in the spelling of the author L. B. Pártay, which was incorrectly given as L. Bartok-Pártay. This has now been corrected in the PDF and HTML versions of the Article, and in the accompanying Supplementary Information file.

