# Supplementary material for: Thermodynamics of CuPt nanoalloys
Source: Sci Rep. 2018 Jun 14;8:9150. doi: 10.1038/s41598-018-27308-1 (PMC6002547; doi:10.1038/s41598-018-27308-1)
Supplement: Supplementary file 1 — Thermodynamics of CuPt nanoalloys [file 41598_2018_27308_MOESM1_ESM.pdf]

Thermodynamics of CuPt nanoalloys

K. Rossi[1,+], L. B. Pártay[2,+], G. Csányi[3], and F. Baletto[1,*]

[1]Physics Department, King's College London, London, WC2R 2LS, UK
[2]Cambridge University, Department of Chemistry, CB2 1EW, Cambridge, United Kingdom, moved to University of Reading, Department of Chemistry, Whiteknights, RG6 6AD, Reading
[3]Cambridge University, Engineering Department, CB2 1PZ, Cambridge, United Kingdom
[*]francesca.baletto@kcl.ac.uk
[+]these authors contributed equally to this work

ABSTRACT

supplementary informations for the original manuscript

1 Computational methods

1.1 Gupta potential, parameters and CuPt parametrization

Gupta potential consists of a repulsive and an attractive term. The first is well approximated by a Born-Meyer term, the latter by a many-body term derived according to the second moment tight binding approximation framework. Considering the local density of states of electrons centered at a specific site and on a certain orbital, the Hamiltonian of the system is given by an atomic level orbital contribution and by an hopping integral. Approximating the local density of states to a rectangular shape, according to a second moment approximation, the attractive term can be found. The aforementioned approximation is well suited for transition metals given the strong d character of their valence states. Parametrization for the different metal-metal interaction according to equation 1 is hereby reported.

According to the Gupta or Rosato-Guillope-Legrande formulation[1,2] the potential, $V_{TBSMA}(r_{ij})$, where r_{ij} is the set of all atomic coordinates, and is evaluated by summing the atomic contributions E^i_{TBSMA}, estimated as in Equation 1. The sum runs over all the atoms n_v within an appropriate cut-off distance from the atom i. a and b refer to the chemical species of the interacting atoms, r^0_{ab} is the bulk nearest neighbor distance for the homo-metallic atomic pairs. It is taken as its arithmetical average for the hetero-metallic case. A_{ab} and ξ_{ab} are related to the cohesive energy, while p_{ab} and q_{ab} tune the stickyness of the potential.

$$E^i_{TBSMA} = \sum_{j\neq i}^{n_v} A_{ab} e^{-p_{ab}\left(\frac{r_{ij}}{r^0_{ab}}-1\right)} - \sqrt{\sum_{j\neq i}^{n_v} \xi^2_{ab} e^{-2q_{ab}\left(\frac{r_{ij}}{r^0_{ab}}-1\right)}} \,. \tag{1}$$

The validation for the parametrization for Cu-Pt interaction used in the manuscript is hereby discussed. Parameters for Pt-Pt and Cu-Cu interaction have been found in the literature and their average was initially used to model Pt-Cu interaction. They are numbered according to their position in Table 1, thus:

1. Cu-Cu[2]Pt-Pt[3]
2. Cu-Cu[2]Pt-Pt[2,4]
3. Cu-Cu[5]Pt-Pt[3]
4. Cu-Cu[5]Pt-Pt[2,4]
5. Cu-Cu[4]Pt-Pt[3]
6. Cu-Cu[4]Pt-Pt[2,4]

Table 1. Resume' of proposed parametrization for Cu-Cu and Pt-Pt interaction as found in the literature..

M-M	A (eV)	p	q	ξ (eV)
Cu-Cu[2]	0.097	11.95	2.27	1.34
Cu-Cu[5]	0.105	11.76	2.27	1.45
Cu-Cu[4]	0.231	10.74	3.87	2.20
Pt-Pt[3]	0.161	10.79	3.14	1.82
Pt-Pt[2,4]	0.175	10.73	3.59	1.79

The sets are then validated against the energy ranking found for density functional theory level of accuracy, of a variety of relaxed structures made of 55 atoms. The architectures considered are clusters of 55 atoms with Cuboctahedron (Co), Icosahedron (Ih), Decahedron (Dh) morphology and Core-shell(cs), Core- Vertex(v), Core (C)-Edge(E)-Vertex(V) impurity chemical compositions. DFT calculations are performed using the Quantum Espresso package[6] using Ultrasoft potential Pardew-Burke-Ernzerhof[?] exchange model within the local spin density approximation. An Energy cutoff = 45.0 eV and a Plane wave cutoff = 360.0A have been used. Calculation sample a single Γ Point in the Brillouin zone.

The parameters proposed in Ref.[7], corresponding to the parametrization number 6, results one of the best performing set as shown by figure 1. In the case of mono-metallic and single impurity systems the energetic ranking is perfectly reproduced. The unique difficulty in reproducing the DFT data is observed for the case of the Pt vertex decorated clusters, hinting to a significant charge transfer in these architectures as also found in the mono-metallic Pt case[8]. Further tuning of the Cu-Pt interaction towards more Cu- or Pt-like interaction in the spirit of Reference[9] didn't result in a significant improvement of the matching between DFT and MD rankings for any of the parameters sets here investigated.

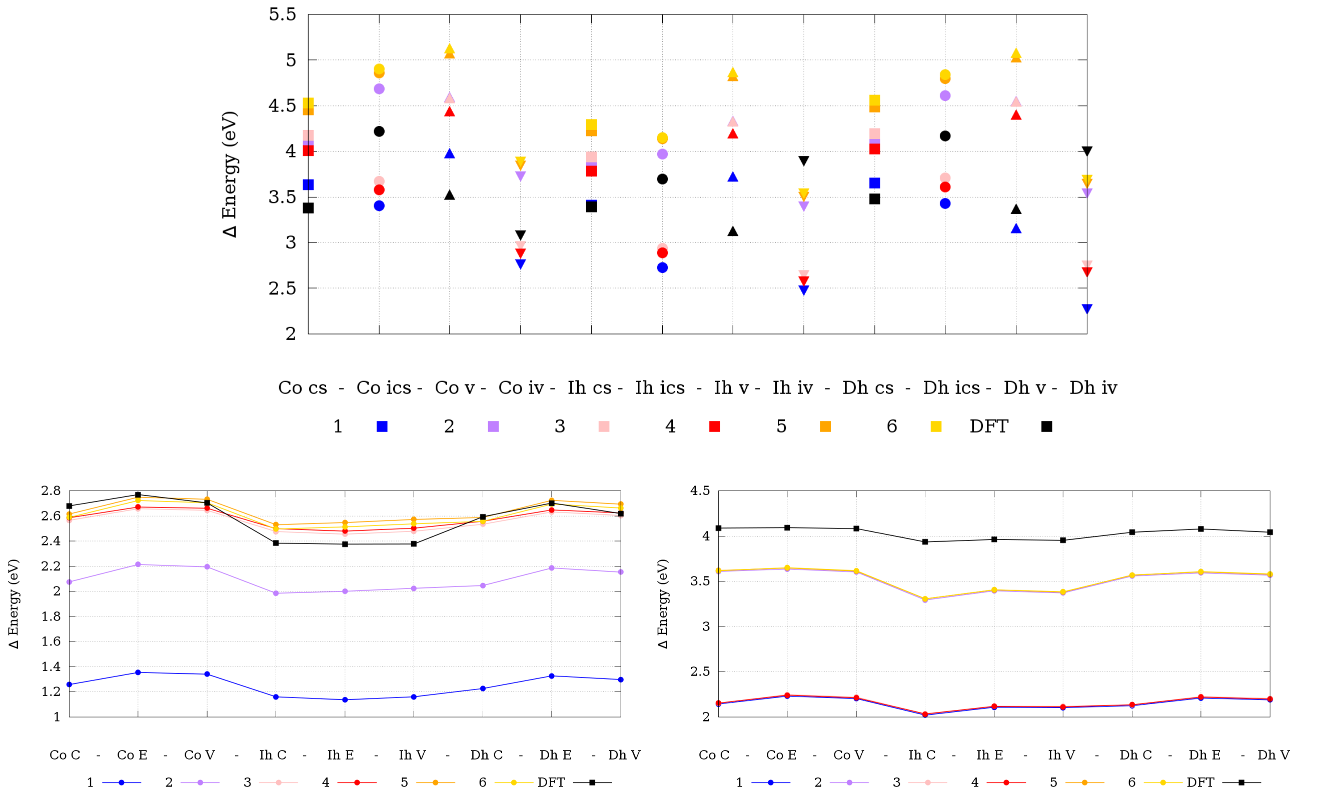

Figure 1. Record of the ΔEnergy of each configuration as calculated through DFT self-consistent level and TBSMA potentials. Energy is rescaled with respect to the bulk cohesion energy contributions. CS and V label refer to a Cu-rich nanoalloy, ICS and IV to a Pt-rich one. Cu_{55} energy ranking is shown on the left, Pt_{55} on the right. TBSMA potentials parameters sets are color coded. Lines are meant as guides to the eyes only.

1.2 Nested Sampling detailed explanation

Nested sampling estimate the density of states of a system by constructing a sequence of decreasing potential energy levels E_i, starting from the vapor phase, each of which bounds from above a volume of configuration space χ_i, with the property that χ_i is approximately a constant factor smaller than the volume χ_{i-1}, corresponding to the level above. Each volume is sampled uniformly, and therefore each distribution will have an approximately constant fractional overlap with the ones immediately before and after, ensuring fast convergence of the sampling and allowing an accurate evaluation of phase-space integrals. In particular, the energy levels near the phase transition, where phase volumes change rapidly, will automatically be very narrowly spaced. The sequence of energy levels comprise a discretization of the cumulative density of states $\chi(E)$, which allows the evaluation of the partition function at arbitrary temperatures,

$$\begin{aligned} Z(N,V,\beta) &= \frac{1}{N!}\left(\frac{2\pi m}{\beta h^2}\right)^{3N/2}\int dE\,\chi'(E)e^{-\beta E} && (2)\\ &\approx Z_m(N,\beta)\sum_i(\chi_{i-1}-\chi_i)e^{-\beta E_i} && (3)\end{aligned}$$

where N is the number of particles of mass m, V is the volume, β is inverse thermodynamic temperature, h is Planck's constant, the density of states χ' is the derivative of χ, and we labeled the factor resulting from the momentum integral as Z_m. In each iteration, we start with a set of samples uniformly distributed and bounded above by energy E_i, and a new energy level is obtained by recording the energy of the sample with highest energy, discarding that sample, and reconstructing the uniform distribution under the new energy limit by cloning a randomly chosen sample from the remaining ones, and de-correlating it from its source sample using a random walk, always obeying the new energy limit. The partition function can be evaluated *a posteriori* at any temperature and other thermodynamic variables, such as the internal energy, can be evaluated as a function of temperature,

$$U(\beta) = -\frac{\partial \ln Z(\beta)}{\partial\beta}. \quad (4)$$

1.3 Iterative molecular dynamics

Molecular Dynamics is a powerful method to integrate the Newton equations of motions and it encodes information on representative trajectories at finite temperatures. The iterative temperature Molecular Dynamics (itMD) consists of concatenated MD runs each of them long $\Delta\tau$, where the temperature in two successive runs is increased by the quantity ΔT. This procedure allows to analysis and report the kinetics of the phase change in terms of a unique parameter, named as temperature change rate, λ which is given by the ratio $\frac{\Delta T}{\Delta \tau}$. itMD allows to estimate caloric curves taking into account potential memory effects at low and high temperatures and it is a more realistic way to mimic the solidification and liquefaction of a system. Furthermore, it allows to detect finite temperature shape transitions whenever their characteristic time is lower than the simulation time at each temperature $\Delta\tau$. As standard output, we report the total energy, potential energy at each instantaneous temperature, as calculated using the equipartition theorem. From them we reconstruct the caloric curve per each simulation. Results are averaged over the set of same time interval (corresponding to same nominal temperature too) runs. Statistical analysis is obtained averaging over

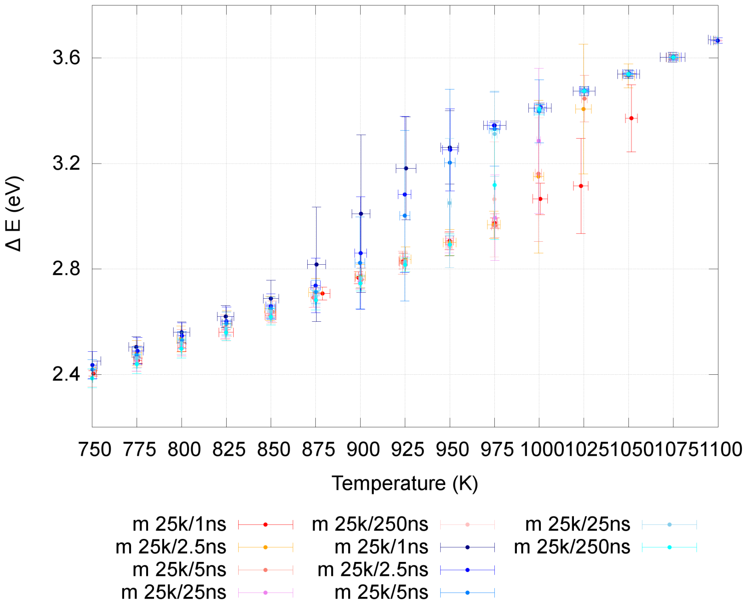

Figure 2. Caloric curve data as obtained from melting and freezing itMD procedures. Each point is averaged over at least 25 independent simulations and the error is the statistical standard deviation. The error on the temperature is too small for being reported.

the various -at least 25- independent itMD simulations performed per each λ value. Figure 2 reports the caloric curves for both melting and freezing procedures. Along the energy axis, each data point is plotted together with its standard deviation. As expected, the statistical error on the energy increases closer to the phase change point, while it is almost negligible in the liquid phase region.

2 Additional examples of cluster taxonomy within our new 2D gIh projection scheme

The mapping of peculiar low-symmetry motif sampled by itMD and Nested sampling according to the 2D giant Icosahedron projection scheme may result not immediate to novel users of this framework. We recap in Figure 3 seven paradigmatic and most common cases of cluster mapping:

a) Cp cluster with all its atoms in an either fcc or hcp arrangement.
b) Dh cluster with its single five-fold symmetry axis, located off-centre.
c) Dh cluster with its single five-fold symmetry axis, located in-centre.
d) Ih cluster with three of its five-fold symmetry axes present and these intersect outside the cluster.
e) Ih cluster where its six five-fold symmetry axes intersect off-centre.
f) Ih cluster with a single five fold symmetry axis near the edge of the cluster, but with an additional sheet of hcp grain boundary at 60° angle indicating the potential positioning of the additional five fold axes and forming an Ih structure. The cluster is also shown from a different angle.
g) Ih cluster with several five-fold symmetry axes, some forming triangles, and one icosahedral centre. An additional snapshot where the white atoms are removed to highlight the five-fold axis triangular arrangements. Dashed lines and circles are guides to eyes.

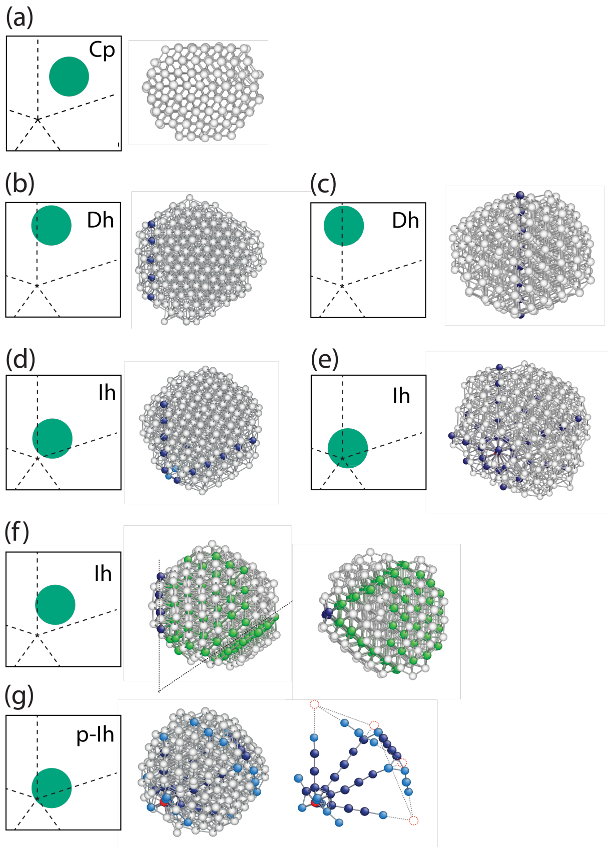

Figure 3. Atoms forming a five-fold symmetry axes are dark blue, the ones forming the incomplete five-fold symmetry axis at the surface of the cluster are light blue, the icosahedral central atom is red, and the hcp atoms are highlighted as green in one case. The rest of the atoms are shown white.

2.1 Caloric curves of CuPt nanoalloys

Caloric curves for the itMD runs with fixed $\Delta\tau$ or fixed ΔT rates and for the NS runs with both 4.8×10^4 and 9.6×10^4 MC steps are shown in Figure ??. The hysteresis loop of the itMD caloric curves gets narrower with slower rates both when fixing ΔT or $\Delta\tau$ in the simulation. The shorter NS runs slightly underestimate the phase transition temperature. The itMD data shown are found by averaging temperature and energies of the system over the ensemble of independent runs with the same temperature change rate.

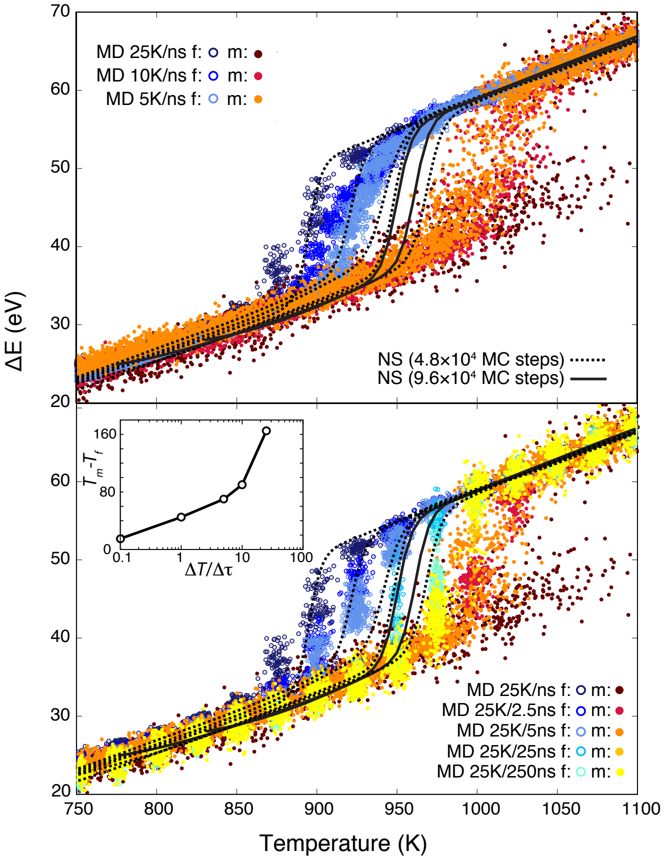

Figure 4. Upper panel: itMD-caloric curves (ΔE is the total energy rescaled with respect to the potential energy of a perfect core-shell icosahedron) with λ equal to 5K/ns, 10K/ns and 25K/ns, shown by light, mid and dark symbols, respectively (hot colours for the melting and cold for freezing). The time interval per each temperature is fixed to 1 ns. For comparison, the NS solidification limits, arising from the seven different set-ups listed in the text, are shown by dashed lines (4.8×10^4 MC steps) and solid black lines (9.6×10^4 MC walk). Bottom panel: itMD-caloric curves at different λ fixing the temperature change at 25 K and varying $\Delta\tau$ from 1 ns (darkest blue/red) to 250 ns (cyan/yellow). In the inset we report the temperature difference between melting and freezing temperatures as a function of λ. The closure of the hysteresis is evident.

2.2 Discussing the nanoalloy's taxonomy during phase changes

Let us first to discuss the taxonomy of CuPt nanoalloys during the exploration with NS. The structural characterization for all seven NS runs are shown in Figure 5. It is clear that NS explores multiple basins in a single run, however in different extent. All NS calculations explored configurations with at least one five-fold symmetry axis, and the icosahedral central atom was always found to be at the very edge of the cluster, except in run III, suggesting that the relative probability of a regular icosahedron is very low. Due to the large size of the cluster and limits in computational resources we had to compromise the sample size used in the calculations. As a result, only two of the runs, II. and VII, explored all four major structural groups and only the results from these two are presented in the manuscript.

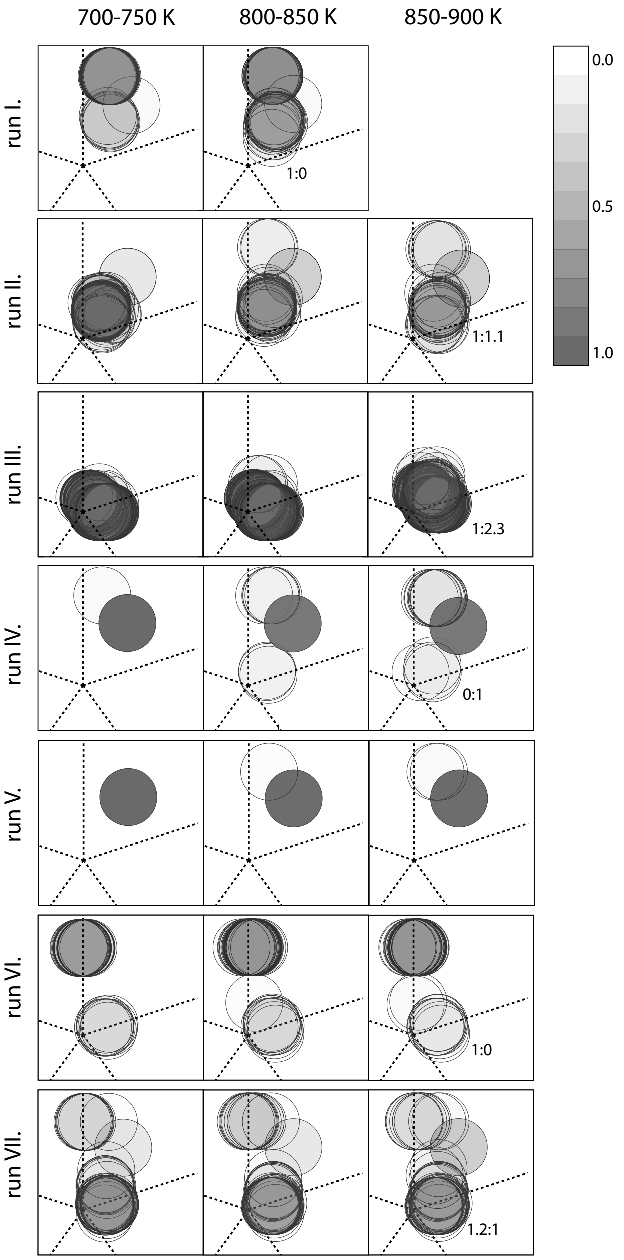

Figure 5. 2D icosahedral projection of the different structural motifs seen in the seven parallel NS calculations (run VI and VII are the runs with the longer MC walk). The columns correspond to different temperature levels, the left one being the closest to the melting transition. Darker shades indicate higher probability and the ratios show the relative ratio of the Ih:p-Ih structures. (Figure for run I at highest temperature is not shown as the freezing occurs below 900 K in this case.)

2.3 itMD clusters taxonomy and morphological transitions analysis

In a similar manner as for NS, we analyze the taxonomy of CuPt during itMD-freezing simulations at different λ rates. Here we illustrate both Ih and p-Ih motifs where the p-Ih label highlights the so called poly-icosahedral clusters, that is, motifs with more than one icosahedral centre, as defined before. In the main text we sum over structures showing a single and more icosahedral centre.

Fig. 6 reports the structural classification of the cluster onto the 2D giant icosahedral map when $\Delta T = 25\ K$ and varying $\Delta\tau$ over two order of magnitude, namely between 1 to 250 ns, at 700 K. We also report in Fig. 7 data for the simulations where the temperature rate ΔT is changed (25K, 10K, and 5K) but we keep fixed the time period between any temperature change, $\Delta\tau$.

itMD was found to explore multiple basins after nucleation only in the case of a freezing rate of 25K/250 ns. In other words, regardless fixing $\Delta\tau$ or ΔT, if the rate λ is larger than 1K/ns, after nucleation, the clusters never undergo any major structural transformations. Structural changes are shown for nine of these runs as a function of temperature in Figure 8. This constitutes an indirect proof that the characteristic timescale for structural transitions below the melting temperature of our system, $Cu_{162}Pt_{147}$, is below 250 ns but much slower than 25 ns.

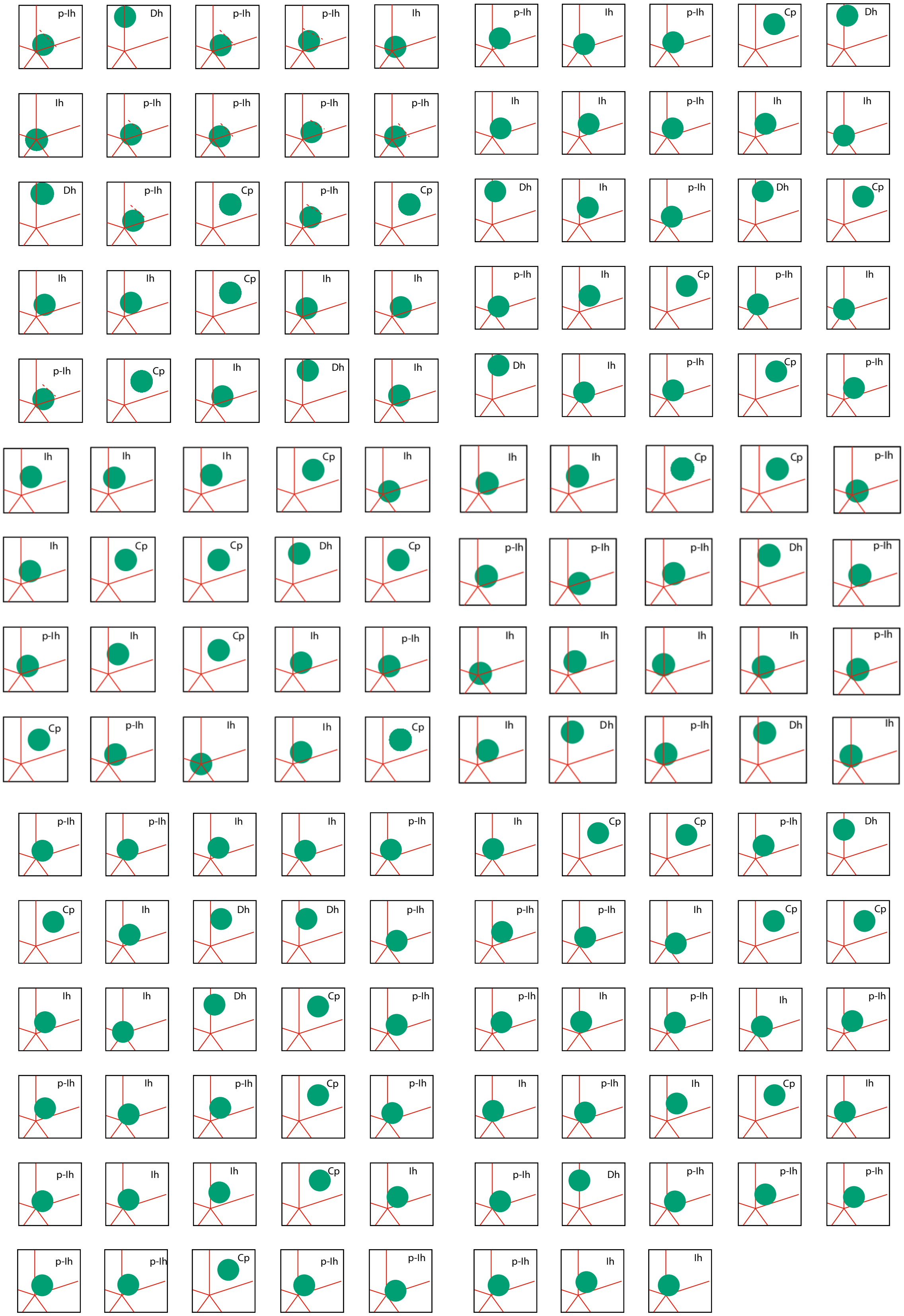

Figure 6. Structural classification of itMD freezing simulations with λ 25 K/ns (top left), 10 K/ns (top right), 5 K/ns (center), 1 K/ns (bottom left), and 0.1 K/ns (bottom right) with $\Delta T = 25\ K$ and varying $\Delta\tau$ accordingly. Each square represents an independent simulation. The green circle shows the nanoparticle shape at 700 K onto the 2D giant icosahedral map represented by red lines, as described in the main text. In the case of $\Delta\tau = 250\ ns$, the green circle refers to a temperature close to 700 K and its cluster evolution is reported in Fig. 8.

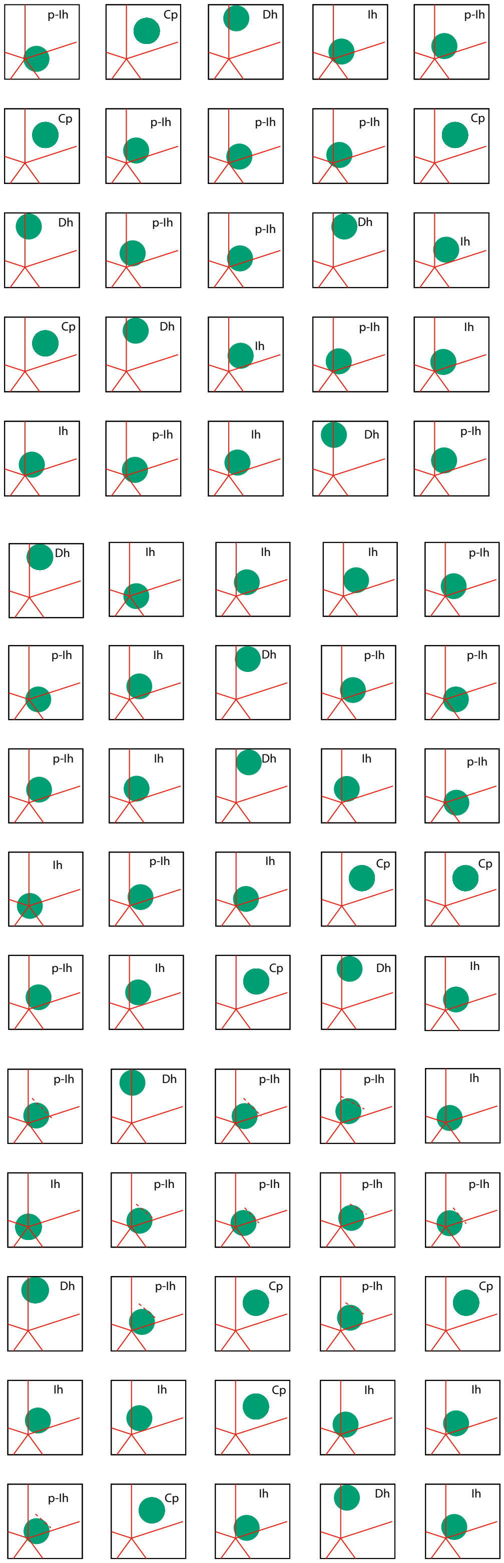

Figure 7. Structural classification of itMD freezing simulations with λ equal to 5 K/ns (left), 10 K/ns (center), and 25K/ns (right), fixing $\Delta\tau = 1$ ns and varying ΔT accordingly. Each square represents an independent simulation. The green circle stands where a structure can be located onto the 2D giant icosahedral map, represented by red lines, at 700 K. The shape name is given in each panel.

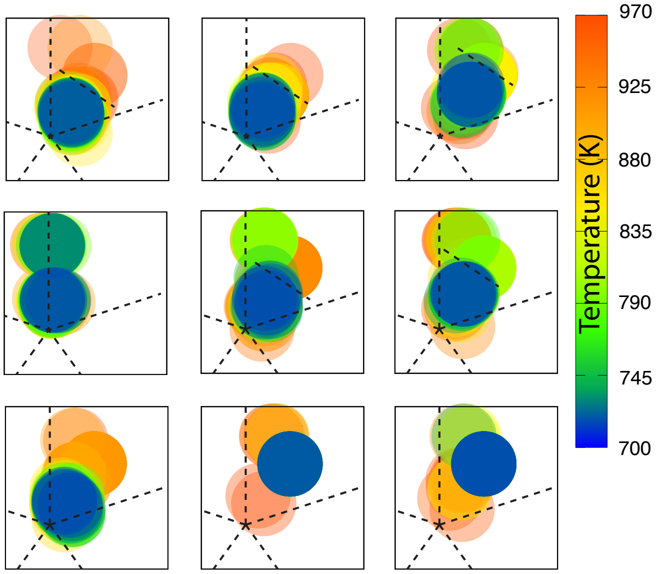

Figure 8. 2D icosahedral projection of the different structural motifs seen in nine of the itMD runs with $\lambda = 0.1\ K/ns$ and $\Delta\tau = 250\ ns$ (runs 1-9 in the bottom right panel of figure 6). Left column: clusters which nucleate into Dh motif and then evolve toward a p-Ih (top), Dh (center), Ih (bot). Middle column: a Cp rearranging as a p-Ih (top), starting as a Cp restructuring into a Dh and then a p-Ih (center), Ih going into a Dh and then a Cp (bot). Right column: a Ih and ending into a p-Ih while going also across a Dh motif (top), a Dh ending into a p-Ih while visiting also the other motifs (center), an Ih rearranging into a Cp after undertaking a Dh geometry. The color coding represents the temperature from hot (red) to cold (blue). Below 700 K, the cluster remains blocked in one shape. For p-Ih the second five-fold axis is reported as dashed line.

3 Order parameters during phase changes

Paradigmatic examples of the temperature evolution of local atomic order parameters such as common neighbor analysis (CNA) signatures and Steinhard parameter Q6 and W6, are depicted in Figure 9, and 10. For the case of CNA signature, paradigmatic bulk signatures are analyzed: the (5,5,5) which detects pair of atoms lying on five-fold symmetry axis, the (4,2,2) which corresponds to pair of atoms along grain boundaries or twinning planes, and the (4,2,1) which characterizes FCC-bulk pairs. For (Q6,W6) pairs, we report specific coordinates corresponding to the ideal value for atoms whose neighbors present an Icosahedral, FCC, HCP, and five-fold symmetric symmetry.

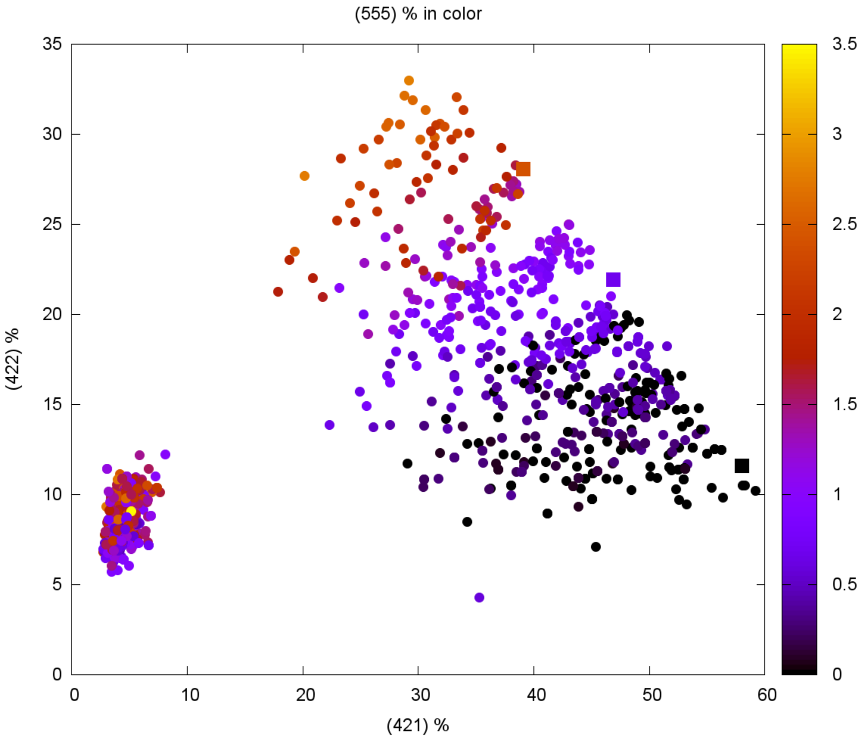

Figure 9. CNA bulk signature (422) and (421) map where the (555) signature percentage is reported in color for structures observed during the set of 25 K/1 ns itMD freezing runs. Melted structures lie in the [0:10 - 5:15] range. Solid structures find their CNA signature occurrence in the [20:60 - 5:35] range instead. On average an higher percentage of (555) signatures are found in the melted cluster with respect to the solid ones.

The solid and the liquid regions are clearly distinguished by a sharp transition of the order parameters under investigation. We note that while the CNA and (Q6,W6) values for the melted phase are representative of the ones found for all the Nested Sampling and itMD runs, their magnitude in the case of a solid structure depend on the morphological basin sampled.

Our NS simulations show an ensemble of liquid, frozen, and half-liquid/half-frozen configurations. Atomic Q6 and W6 parameters are also reported to distinguish solid and liquid atoms according to the mapping shown in Figure 10

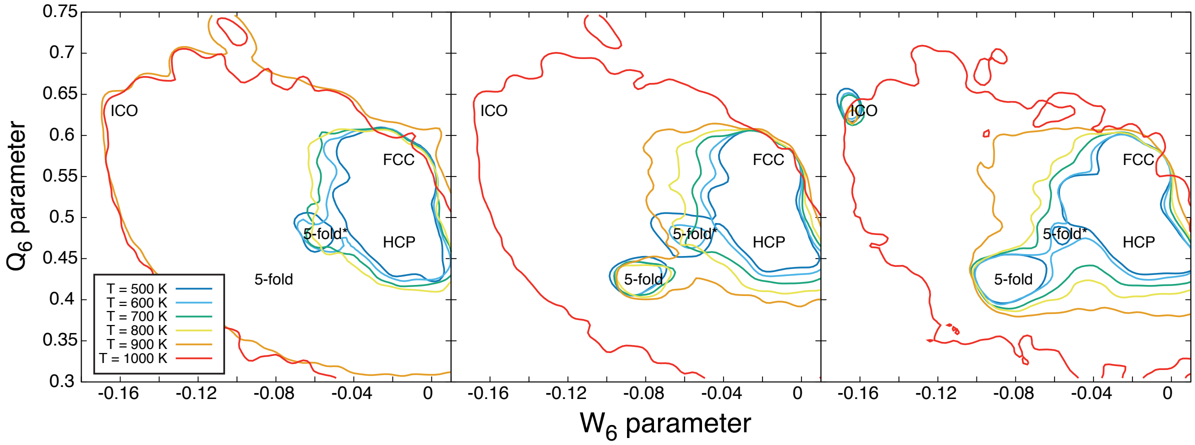

Figure 10. Q6 and W6 map where iso-probable regions corresponding to different temperatures are highlighted in colors. Data are gathered from three independent NS runs. Also in this case note the correspondence between the nucleation of solid structure and the clustering of the Q6 and W6 values around characteristic values peculiar of local atomic environments in nanoclusters. The ICO, 5-fold(*), HCP, and FCC label refer to characteristic (Q6,W6) values for icosahedral centres, atoms in a (in)complete five-fold axis, or in FCC and HCP sheets respectively.

4 Chemical reordering in CuPt nanoalloys

To check if chemical re-ordering might happen on a time scale similar to shape transitions, we run additional itMD melting simulations starting from unfavorable chemical ordering, for example, a segregated one as in the case of a core-shell. Fig 11 shows the time evolution of the radial chemical distribution function of a $Cu_{162}Pt_{147}$ cluster with an initial Pt core - Cu shell at 300K, 600K, and 900K. Within the time scale probed in this simulation, 100 ns , the chemical ordering is preserved for the two lower temperatures, and strongly altered, leading to the formation of the favorable onion-shell pattern, only for the 900 K run.

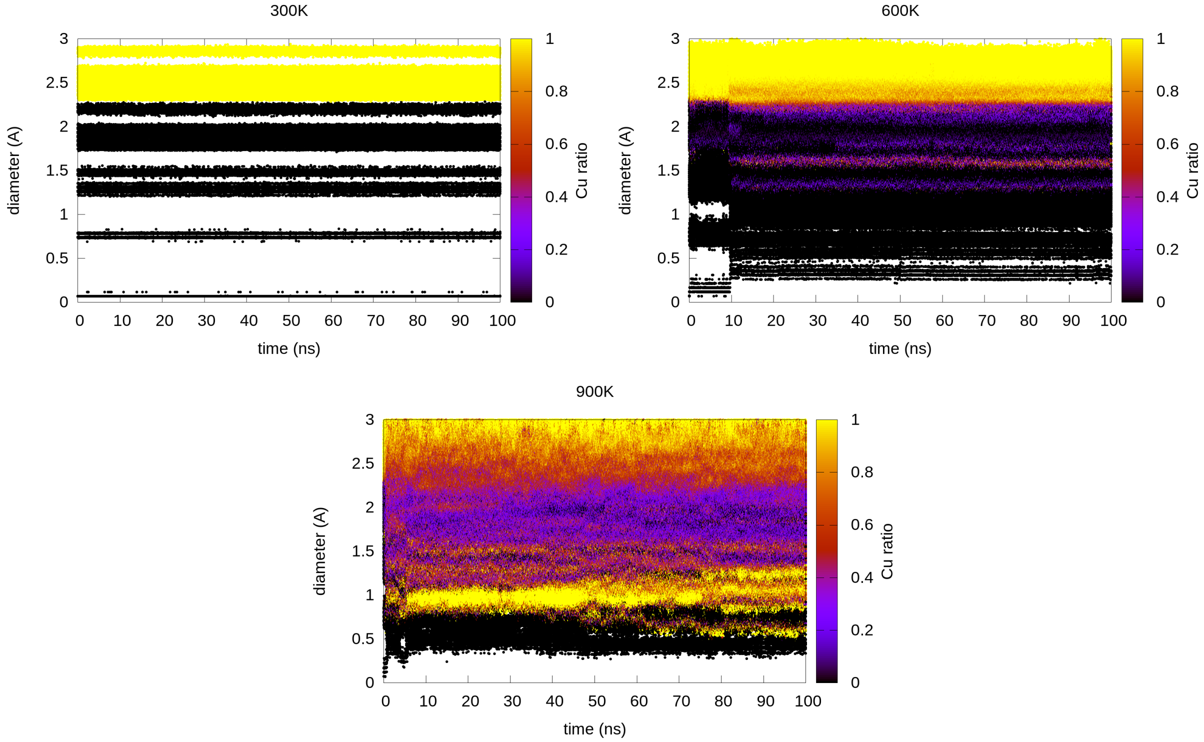

Figure 11. Time evolution of the ratio of the Cu atoms within different 0.01A thick layers of a $Cu_{162}Pt_{147}$ cluster at 300K, 600K, and 900K when starting from a perfect icosahedral morphology and a Pt core - Cu shell chemical ordering.

References

1. Rosato, V., Guillope, M. & Legrand, B. Thermodynamical and structural properties of f.c.c. transition metals using a simple tight-binding model. *Philos. Mag. A* **59**, 321–336 (1989). DOI 10.1080/01418618908205062.

2. Cleri, F. & Rosato, V. Tight-binding potentials for transition metals and alloys. *Phys. Rev. B* **48**, 22–33 (1993). URL https://link.aps.org/doi/10.1103/PhysRevB.48.22. DOI 10.1103/PhysRevB.48.22.

3. Cheng, D., Yuan, S. & Ferrando, R. Structure, chemical ordering and thermal stability of Pt–Ni alloy nanoclusters. *J. Physics: Condens. Matter* **25**, 355008 (2013). URL http://stacks.iop.org/0953-8984/25/i=35/a=355008?key=crossref.0a8a2097ba39cba2c98db8fdfe36faed. DOI 10.1088/0953-8984/25/35/355008.

4. Baletto, F., Ferrando, R., Fortunelli, A., Montalenti, F. & Mottet, C. Crossover among structural motifs in transition and noble-metal clusters. *J. Chem. Phys.* **116**, 3856–3863 (2002). URL http://aip.scitation.org/doi/10.1063/1.1448484http://scitation.aip.org/content/aip/journal/jcp/116/9/10.1063/1.1448484. DOI 10.1063/1.1448484.

5. Panizon, E., Olmos-Asar, J. A., Peressi, M. & Ferrando, R. Study of structures and thermodynamics of cuni nanoalloys using a new dft-fitted atomistic potential. *Phys. Chem. Chem. Phys.* **17**, 28068–28075 (2015). DOI 10.1039/C5CP00215J.

6. Giannozzi, P. *et al.* QUANTUM ESPRESSO: a modular and open-source software project for quantum simulations of materials. *J. Physics: Condens. Matter* **21**, 395502 (2009). URL http://stacks.iop.org/0953-8984/21/i=39/a=395502?key=crossref.c21336c286fa6d3db893262ae3f6e151. DOI 10.1088/0953-8984/21/39/395502.

7. Pavan, L., Baletto, F. & Novakovic, R. Multiscale approach for studying melting transitions in cupt nanoparticles. *Phys. Chem. Chem. Phys.* **17**, 28364–28371 (2015). URL http://dx.doi.org/10.1039/C5CP01096A. DOI 10.1039/C5CP01096A.

8. Di Paola, C., D'Agosta, R. & Baletto, F. Geometrical Effects on the Magnetic Properties of Nanoparticles. *Nano Lett.* **16**, 2885–2889 (2016). URL http://pubs.acs.org/doi/abs/10.1021/acs.nanolett.6b00916. DOI 10.1021/acs.nanolett.6b00916.

9. Paz-Borbón, L. O., Gupta, A. & Johnston, R. L. Dependence of the structures and chemical ordering of Pd–Pt nanoalloys on potential parameters. *J. Mater. Chem.* **18**, 4154 (2008). URL http://xlink.rsc.org/?DOI=b805147j. DOI 10.1039/b805147j.
